# Editorial comment: Clinical Recommendations From the European Society for Sexual Medicine Exploring Partner Expectations, Satisfaction in Male and Phalloplasty Cohorts, the Impact of Penile Length, Girth and Implant Type, Reservoir Placement, and the Influence of Comorbidities and Social Circumstances

**DOI:** 10.1590/S1677-5538.IBJU.2021.03.06

**Published:** 2021-03-25

**Authors:** Valter Javaroni

**Affiliations:** 1 Hospital Federal do Andaraí Departamento de Andrologia Rio de JaneiroRJ Brasil Departamento de Andrologia, Hospital Federal do Andaraí, Rio de Janeiro, RJ, Brasil

Daniar Osmonov^1^, Andrew Nim Christopher^2^, Gideon A Blecher^3^, Marco Falcone^4^, Armin Soave^5^, Roland Dahlem^5^, et al.

J Sex Med. 2020 Feb;17(2):210-237.

## COMMENT

Guidelines for Male Sexual Dysfunction recognize penile prosthesis implantation as the third line treatment for erectile dysfunction (ED). Given the invasive and essentially irreversible nature of penile prosthesis implantation (PPI) surgery, counseling regarding short- and long-term postoperative expectations is essential (1).

Since the beginning of the penile prosthesis implantation, multiple modifications have been made which has lead to decreased postoperative infection and mechanical failure and increased rigidity, durability, and patient satisfaction (2,3).

Currently, the preferred type of penile implant in developed countries is the inflatable one. In certain regions of the world (like Brazil), the malleable penile prosthesis is the penile implant of choice for ED, certainly because of economic reasons (no insurance coverage) (4), but providing high patient and partner satisfaction rates (5).

In this recent publication, the European Society for Sexual Medicine (ESSM) warns that a majority of the studies published on PPI deal with clinical or technical aspects of surgery, but not with associated factors, such as the patients’ and partners’ expectations, comorbidities, and social profiles. Over the last decades, a number of articles have described the expectations of both patients and their partners, the influence of the patients’ comorbidities, as well as a variety of social aspects in association with penile prosthesis. But most publications derived from single center, retrospective studies with low patient numbers and limited follow-up.

There is a need for appropriate patient selection and counseling with any surgical procedure, particularly when quality of life and satisfaction are the primary objectives of treatment. Despite the unavoidable nature of complications in general, the ability to discriminate appropriate surgical candidates remains completely within the control of the surgeon (6).

The satisfaction of the patient and the partner is the most important end point of PPI. It is increasingly relevant to assess the psychosocial status of the couple and to inform them about the procedure to avoid unrealistic expectations (5).

Satisfaction with IPP is influenced by many factors, which can be categorized into three distinct patient care stages: preoperative, intraoperative, and postoperative. There are several techniques described in each patient care stage to improve perceived penile length including preoperative counseling and correct penile length measuring, preoperative penile stretching, the use of adjuvant surgical procedures, and postoperative penile rehabilitation (7).

The CURSED (mnemonic CURSED Patient: “Compulsive/obsessive, Unrealistic, Revision, Surgeon Shopping, Entitled, Denial, and Psychiatric) assessment of preoperative expectations can help to identify high- risk patients, a challenging subset of patients that may be at increased risk of postoperative dissatisfaction. Character traits of those difficult patients include obsessive/ compulsive tendencies, unrealistic expectations, those seeking multiple surgical options, feelings of entitlement, patients in denial of their prior erectile/sexual function and current disease status, or those with other psychiatric disorders (8).

This ESSM position statement provides relevant recommendations on optimization of patient outcome by patient selection, and individualized peri- and intra-operative management. The article clarifies the multiple aspects of PPI surgery, offering an evidence-based clinical framework to guide patient-tailored management of ED. Influence of comorbidities and social circumstances like smoking, obesity, aging, H.I.V., organ transplantation, spinal cord injury, Peyronie disease and diabetes; The importance of a thoroughly discussion on expected post-operative outcomes with both partners prior to PPI surgery, including possible complications and their management; The impact of length, girth, and implant type on satisfaction; And even IPP in transgender gender, are all addressed during this nice review.

According to ESSM, the influence of cultural and social factors related to the aging process and a shift in the expression of sexuality due to increased life expectancy, as well as improvement of quality of life over the last decade, increases the role of sexual medicine.

The authors concluded that standardized methods for assessment of patient and partner expectations have not been established resulting in heterogeneous study types that evaluate different outcomes and have a low (<3) level of evidence. Many studies used validated scoring systems, such as EDITS or IIEF. However, these have not necessarily been developed specifically for the evaluation of penile implant outcomes. Validated scoring systems for PPI surgery include the Quality of Life and Sexuality with Penile Prosthesis (9).

And their final message stresses the need for larger prospective multicentric epidemiological studies that should be initiated and ideally supported by international societies.
